# Low Urinary Free Cortisol as a Risk Factor for Patients with Variceal Bleeding

**DOI:** 10.3390/medicina59122112

**Published:** 2023-12-02

**Authors:** Ioanna Aggeletopoulou, Efthymios P. Tsounis, Maria Kalafateli, Maria Michailidou, Maria Tsami, Konstantinos Zisimopoulos, Martha Mandellou, Georgia Diamantopoulou, Maria Kouskoura, Marina Michalaki, Catherine K. Markopoulou, Konstantinos Thomopoulos, Christos Triantos

**Affiliations:** 1 Division of Gastroenterology, Department of Internal Medicine, University Hospital of Patras, 26504 Patras, Greece; iaggel@upatras.gr (I.A.); up1081417@upatras.gr (E.P.T.); mariakalaf@hotmail.com (M.K.); marmicpan@hotmail.com (M.M.); condoczisimo@hotmail.com (K.Z.); geodiamant@hotmail.com (G.D.); kxthomo@hotmail.com (K.T.); 2 Laboratory of Pharmaceutical Analysis, Department of Pharmaceutical Technology, School of Pharmacy, Faculty of Health Sciences, Aristotle University of Thessaloniki, 54124 Thessaloniki, Greece; tsammari@pharm.auth.gr (M.T.); mkousk@pharm.auth.gr (M.K.); amarkopo@pharm.auth.gr (C.K.M.); 3 Department of Biochemistry, University Hospital of Patras, 26504 Patras, Greece; mandelloum@gmail.com; 4 Division of Endocrinology, Diabetes and Metabolic Diseases, Department of Internal Medicine, University of Patras, 26504 Patras, Greece; mixmar@upatras.gr

**Keywords:** 24 h urinary free cortisol, liver cirrhosis, decompensation, 6-week mortality, variceal bleeding, 5-day treatment failure, 24 h UFC

## Abstract

*Background and Objectives:* Specificity and reliability issues of the current cortisol assessment methods lead to limitations on the accurate assessment of relative adrenal insufficiency. Although free cortisol provides a more accurate evaluation of adrenal cortisol production, the expense and time-consuming nature of these assays make them impractical for routine use. Research has, thus, focused on alternative methods, such as indirectly measuring free cortisol using Coolens’ equation or directly assessing salivary cortisol concentration, which is considered a more favorable approach despite associated challenges like sampling issues and infection risks. The aim of this study was to explore correlations between 24 h urinary free cortisol (UFC), free plasma cortisol, serum total cortisol, and salivary cortisol as potential reliable indices of free cortisol in the setting of variceal bleeding. Additionally, we assessed the predictive value of UFC for 6-week mortality and 5-day treatment failure in patients with liver cirrhosis and variceal bleeding. *Materials and Methods:* A total of 40 outpatients with liver cirrhosis and variceal bleeding were enrolled. Free cortisol levels in serum, saliva, and urine were assessed using the electrochemiluminescence immunoassay method. For the measurement of plasma-free cortisol, a single quadrupole mass spectrometer was employed. The quantification of free cortisol was fulfilled by analyzing the signal response in the negative ESI-MS mode. *Results:* UFC was significantly correlated to free plasma cortisol. Negative correlations were demonstrated between UFC, the Child–Pugh (CP) score, and C reactive protein (CRP) levels. In the multivariate analysis, CP stage C was associated with 6-week mortality risk and portal vein thrombosis with 5-day treatment failure using Cox regression and binary logistic regression analyses, respectively. Patients who experienced rebleeding, infection, or death (or any combination of these events) presented with lower levels of UFC. *Conclusions*: This study suggests that low levels of UFC may impose a risk factor for patients with liver cirrhosis and variceal bleeding. The use of UFC as an index of adrenal cortisol production in variceal bleeding warrants further investigation.

## 1. Introduction

Approximately half of patients with liver cirrhosis present with esophageal varices at diagnosis [1], with higher incidence rates in decompensated compared to compensated cirrhotic patients (30–40% vs. up to 85%, respectively) [2]. Acute variceal bleeding (AVB), a major complication of portal hypertension, constitutes a life-threatening condition and is associated with high mortality rates [2,3,4,5]. Patients surviving the first variceal bleeding episode present an augmented risk for recurrence, mainly during the first 6-weeks [5].

Relative adrenal insufficiency (RAI) is observed during critical illness in individuals with an apparently normal hypothalamic–pituitary axis (HPA) axis. More precisely, the amount of cortisol production is inadequate for the severity of the illness, leading to functional insufficiency [6]. This entity is mostly described in sepsis and septic shock. is the pathogenesis of RAI is largely unknown, and it could be attributed to aberrant cytokine production, low lipoprotein cholesterol levels, which serve as a substrate for cortisol production, and drug interactions [6,7]. Ιn patients with liver cirrhosis, the presence of RAI, known as hepato-adrenal syndrome, has been highlighted as a major clinical issue [8]. RAI was initially described in cirrhotic patients with severe sepsis or septic shock [9,10,11]; however, further studies showed that RAI was also present in non-critically ill cirrhotic patients [8,11,12,13,14,15,16,17,18] and patients with decompensation other than sepsis, such as AVB [19,20].

During AVB, a markedly reduced adrenocortical response to stress is documented; this response may be unsuitable for the degree of illness severity [21] and is possibly associated with worse bleeding-related outcomes in cirrhosis with RAI compared to those without RAI [19]. In our previous study, increased calculated free cortisol levels were found to be predictive of short-term bleeding-related mortality in AVB [20].

Existing limitations related to the specificity and reliability of the available cortisol measurement methods lead to the insufficient assessment of RAI. Free cortisol, which is the biologically active hormone [22], offers a more accurate evaluation of adrenal cortisol production, especially in patients with abnormal cortisol-binding globulin (CBG) levels [23]. However, the use of free cortisol for the evaluation of RAI is quite difficult, as cortisol secretion is dependent on the patient’s physical and mental state and stress conditions and presents a circadian rhythm with alterations over a 24 h period [24]. In addition, assays that measure plasma-free cortisol are costly and time-consuming, making them unsuitable for routine use, particularly in urgent clinical situations. Total serum cortisol is approximately 70% bound to CBG, which is synthesized in the liver, 20% bound to albumin, and 10% is free (unbound) [25,26,27,28,29]. In the setting of decreased concentrations of CBG or albumin, the serum total bound cortisol is reduced while there is no change in the unbound fragment. In this setting, serum total cortisol levels are not in equilibrium with free cortisol levels [25,26]. Thus, total serum cortisol levels have been considered problematic for the determination of adrenal cortisol production in patients with liver cirrhosis, especially in seriously ill patients [7,30]. Hence, research has been focused on alternative methods, such as indirectly measuring free cortisol using Coolens’ equation or directly assessing salivary cortisol concentration, which is considered a more favorable approach [31]. Salivary cortisol determination is an easily available and more accurate method to assess adrenal function in patients with cirrhosis, hypoalbuminemia, or CBG depletion [12,32,33,34,35,36,37]. However, salivary cortisol has certain limitations, including issues related to sampling in intubated and dehydrated patients [38]. Moreover, there is a risk for bacterial or candida infections in the oral cavity, the contamination of saliva with blood due to minor injuries, and the possibility of blood leakage in the oral cavity, particularly in patients with poor oral hygiene or variceal bleeding. These factors can influence the measured concentrations of cortisol in saliva [38,39].

In physiological states, around 10% of serum cortisol is unbound and biologically active. Most of the unbound cortisol is reabsorbed by the renal tubules; however, a minor fraction, approximately 1%, is excreted and remains unchanged in urine. Consequently, the measurement of 24 h UFC directly evaluates the presence of circulating free cortisol and is not influenced by conditions that influence CBG levels [40]. The assessment of 24 h UFC excretion has been frequently used for the evaluation of HPA axis function [41,42,43]. Utilizing 24 h urinary free cortisol (UFC) offers the advantage of being impervious to short-term cortisol fluctuations and variations in plasma protein binding capacities [44]. It is considered a reflection of daily cortisol production and is relatively convenient to collect in large study populations, especially when compared to serial blood sampling [45]. In our recent study, we showed a significant association between 24 h UFC with serum-free cortisol in patients with stable liver cirrhosis, highlighting its use as a reliable index of adrenal function [46]. Moreover, 24 h UFC was found to be predictive of cirrhotic patients’ survival [46]. Considering that the early prognosis of adrenal insufficiency is crucial for cirrhotic patients [14], as it is considered an early survival predictive factor [47] and that patients with AVB present a high risk for 6-week mortality, evidence of adrenal dysfunction on the basis of 24 h UFC and its possible associations with adverse outcomes is essential.

The aim of the present study was to examine the potential correlations between 24 h UFC levels and free plasma cortisol, serum total cortisol, and salivary cortisol and to evaluate their use as reliable markers of true cortisol in the setting of variceal bleeding. The secondary aim was the evaluation of 24 h UFC as a prognostic marker of 6-week mortality and 5-day treatment failure in patients with liver cirrhosis and variceal bleeding.

## 2. Materials and Methods

### 2.1. Study Population

Caucasian patients with liver cirrhosis and variceal bleeding were consecutively enrolled in this prospective observational study. This study was conducted in the Division of Gastroenterology, Department of Internal Medicine, University General Hospital (PUH) of Patras, Patras, Greece. Patients’ follow-up was performed at regular intervals according to current guidelines [2,48] until liver transplantation, the completion of the study, or death. The exclusion criteria were age < 18, pregnancy, a history of hypothalamic-pituitary or adrenal disease, the use of corticosteroid treatment within the past 6 months, HIV infection, hepatocellular carcinoma (HCC) beyond the Milan Criteria, septic shock or severe sepsis, albumin or fresh frozen plasma administration and the refusal of participation in the study.

### 2.2. Definitions

Liver cirrhosis and decompensation were diagnosed based on clinical, histological, laboratory, and ultrasonographic findings [49,50,51]. Severity of cirrhosis was assessed by the Child-Pugh (CP) stage and the Model for End-stage Liver Disease (MELD) score [52,53]. Variceal bleeding was diagnosed based on hematemesis or melena with either a bleeding varix (active bleeding or a clot adherent to the varix or variceal ulceration) or the absence of another bleeding source during upper gastrointestinal endoscopy. Severe bleeding was defined as arterial hypotension (systolic blood pressure < 100 mm/Hg) or/and hemoglobin < 8 g/dL at admission. The primary outcome assessed in the current study was 6-week mortality according to the expanding consensus in the portal hypertension report of the Baveno VII workshop [54]. The secondary outcome assessed in this study was a 5-day treatment failure; this outcome was defined either by the absence of bleeding control or by rebleeding within the first 5 days after the bleeding episode [54].

### 2.3. Data Collection

The demographic characteristics, the medical history, the etiology of liver cirrhosis, and the first episode of variceal bleeding or recurrence were recorded.

### 2.4. Urine and Saliva Sample Preparation

Early morning peripheral blood samples (3–4 mL) were collected using serum separator tubes. The samples were centrifuged, and the sera were obtained and used for serum total cortisol determination. Urine samples were collected over a 24 h period for the measurement of 24 h UFC levels. Saliva samples were obtained to determine saliva cortisol levels.

### 2.5. Cortisol Determination

#### 2.5.1. Serum Cortisol, 24 h UFC and Salivary Cortisol Determination

Serum cortisol, 24 h UFC and salivary cortisol levels were assessed using fully automated highly sensitive competitive electrochemiluminescence (ECLIA) immunoassays using the Elecsys Cortisol reagent kit, Cobas, Roche Diagnostics (Lot number 00711650) on the MODULAR ANALYTICS Ε170 analyzer (Roche Diagnostics, GmbH, Mannheim, Germany). The measuring range was 0.5–1750 nmol/L or 0.054–63.4 µg/dL. The functional sensitivity (lower limit of quantification) of the assay was <8.5 nmol/L (<0.308 μg/dL). The cross-reactivities of this antibody were as follows: corticosterone (5.8%), cortisol-21-sulfate (0.04%), cortisone (0.30%), 11-deoxycorticosterone (0.69%), 11-deoxycortisol (4.1%), dexamethasone (0.08%), 17-α-hydroxyprogesterone (1.50%), prednisone (0.28%), progesterone (0.35%).

#### 2.5.2. Serum-Free Cortisol Determination

Serum-free cortisol was determined using liquid chromatography-electrospray ionization mass spectrometry (LC–ESI–MS) coupled with a quadrupole analyzer (LC-MS, 2020; Shimadzu, Tokyo, Japan). Τhe mobile phase, consisting of acetonitrile and a 0.1 N aqueous formic acid solution (50% *v*/*v*), was eluted isocratically (flow rate 0.5 mL/min) into a Supelcosil LC-18-DB: C_18_ column. The voltage of the detector and interface was 1.00 kV and −3.5 kV, respectively. The nebulization gas flow was 1.5 L/min, and that of the drying gas was 15 mL/min. The temperature of the desolvation line was set at 250 °C, and that of the thermal block was 200 °C. The LC-MS-2020 operated in the negative ionization SIM mode, targeting two ions *m*/*z* = 407 for cortisol and *m*/*z* = 447 for prednisolone acetate (internal standard). The *m*/*z* signals were associated with adduct ions resulting from formic acid. The elution time was 7 min for cortisol and 10.5 min for prednisolone acetate. For the quantification of free cortisol, three calibration curves at 6 different concentrations of 4.32–345.6 ng/mL were prepared. The first was made with known concentrations of cortisol and prednisolone acetate (internal standard); in the second, the same standard solutions were subjected to solid phase extraction (SPE) treatment prior to analysis, while in the third curve, the standard solutions were spiked on a serum substrate desalted from the two analytes (by SPE technique), which were then re-submitted to SPE. This systematic approach ensured the possibility of quantifying the samples using all three reference curves since their slope provided similar values.

#### 2.5.3. SPE Protocol

Prior to analysis, samples were stored in polypropylene vials at −80 °C. They were cleaned up through an SPE protocol using Bond Elut C18 500 mg, 6 mL cartridges. Acetonitrile, a 0.1 N aqueous formic acid solution, and water were used to perform the SPE procedure as follows: The column was initially conditioned with 2 mL of acetonitrile (ACN) and 1 mL of 0.1 N formic acid (HCOOH 0.1% *v*/*v*). This was followed by the loading of the mixture of interest (250 μL of hydrocortisone solution of a specific concentration + 250 μL of prednisolone acetate solution + 500 μL of water in the case of unspiked standards or serum in the case of spiked standards) or 1 mL of the sample. For column wash, 1 mL of the ACN:HCOOH mixture at a ratio of 10–90% and 2 mL of water (H_2_O) was applied. Before the final stage of the SPE process, the waste resulting from the previous steps was discarded. Finally, the elution and recovery of the analytes of interest was performed by applying 1 mL of ACN. The flow rate at each stage was approximately 1 drop per 3–4 s.

#### 2.5.4. CBG Determination

CBG was assessed using an enzyme-linked immunosorbent assay in compliance with the manufacturer’s protocol (product #RD192234200R; BioVendor, LLC, Asheville, NC, USA).

### 2.6. Statistical Analysis

Numerical variables were expressed as medians and interquartile ranges (IQR), and categorical variables were expressed as counts (percentages). Continuous variables were not normally distributed according to Kolmogorov–Smirnov’s test; therefore, an analysis was performed using non-parametric tests. The Mann–Whitney U test was used to compare the medians between the two groups. Spearman’s correlation coefficient was used to evaluate the degree of association between two variables. Pearson’s chi-square test or Fisher’s exact test, when applicable, were used for comparison between categorical data. Univariate and multivariate Cox regression analysis were used to define independent predictors for 6-week mortality post-variceal bleeding. Univariate and multivariate binary logistic regression analyses were used to assess independent predictive factors for variceal rebleeding. All variables with a *p*-value of <0.1 in the univariate analysis were first included in the multivariate models and then eliminated using backward selection. All statistical tests were two-sided. The SPSS statistical package (version 28.0, IBM, New York, NY, USA) was used.

### 2.7. Ethics

All study participants or their legal guardians provided written informed consent prior to enrollment in the study. The study protocol was reviewed and approved by the Ethics Committee Scientific and by the Review Board of the University Hospital of Patras, Greece, with approval number [289/23.07.2014]. The study protocol adheres to the ethical guidelines of the 1975 Declaration of Helsinki for medical research involving human subjects. All the experiments were performed as specified by relevant regulations and guidelines of the concerned ethics committee.

## 3. Results

The main baseline demographic, biochemical, and clinical characteristics of the patients are presented in Table 1.

Overall, 40 patients with liver cirrhosis and variceal bleeding were enrolled in the study. The study population was followed up over a period of 6 weeks. The adrenal function parameters of the study population are summarized in Table 2.

### 3.1. Correlations between Total Serum Cortisol, Free Plasma Cortisol, Salivary Cortisol, CBG, and 24 h UFC

Total serum cortisol was significantly correlated to salivary cortisol (r = 0.356, *p* = 0.049) and free plasma cortisol levels (r = 0.672, *p* < 0.001). CBG was significantly related to free plasma cortisol levels (r = 0.449, *p* = 0.009). The 24 h UFC was significantly associated with free plasma cortisol levels (r = 0.362, *p* = 0.042). The associations between the various cortisol forms are presented in Supplementary Table S1.

### 3.2. Correlations between Total Serum Cortisol, Free Plasma Cortisol, Salivary Cortisol, CBG, and 24 h UFC and Clinical Parameters

In Supplementary Table S2, the associations between the different cortisol forms and various clinical parameters are presented. The total serum cortisol presented a significant negative association with total bilirubin levels (r = −0.358, *p* = 0.038), the salivary cortisol presented negative relation with albumin levels (r = −0.472, *p* = 0.007), and lastly, the 24 h UFC presented significant negative correlations with CP score (r = −0.360, *p* = 0.047) and with C reactive protein (CRP) levels (r = −0.439, *p* = 0.041).

### 3.3. 6-Week Mortality

The 6-week overall mortality rate was 10% (4 out of 40 patients). The factors that were significantly associated with 6-week mortality were assessed using Cox regression analysis and are presented in Supplementary Table S3. In the univariate analysis, portal vein thrombosis (*p* = 0.043), the CP stage C (*p* = 0.029), the development of in-hospital infection (*p* = 0.043), and the presence of rebleeding (*p* = 0.007) were found to be significantly associated with the 6-week mortality risk. CBG levels (*p* = 0.065) and the CP score (*p* = 0.06) were marginally related to this risk. In the multivariate analysis, only CP stage C was found to be independently predictive for 6-week mortality (HR 27.00, 95% CI 1.26–578.35, *p* = 0.035).

### 3.4. 5-Day Treatment Failure

The 5-day treatment failure rate was 5% (2 out of 40 patients). Binary logistic regression analysis was applied to determine the 5-day treatment failure-associated factors. The results are presented in Supplementary Table S4. In the univariate analysis, portal vein thrombosis (*p* = 0.004) and CP stage C (*p* = 0.05) were significantly associated with the 5-day treatment failure risk. This outcome was also marginally associated with CBG levels (*p* = 0.06) and the CP score (*p* = 0.06). In the multivariate analysis, only portal vein thrombosis was found to be significantly associated with this outcome (OR 39.00, 95% CI 2.67–569.67, *p* = 0.007).

### 3.5. Risk for Treatment Failure, Infection or Mortality

During the follow-up period, 4 (10%) patients experienced rebleeding, 4 (10%) patients developed infection, and 4 (10%) patients died. Patients who rebled, developed an infection, died during follow-up, or had any combination of these events were reported as having a composite endpoint, and this endpoint was observed in 9 patients (22.5%). A comparison of cortisol concentrations between patients who experienced rebleeding, infection, or death (or any combination of these events) versus those who remained free of these events showed that the first group presented lower levels of 24 h UFC compared to the latter (Supplementary Table S5).

## 4. Discussion

Τhe present study is the first to establish a correlation between 24 h UFC levels and plasma-free cortisol levels in patients with liver cirrhosis and variceal bleeding, indicating its potential use as a reliable indicator of adrenal cortisol production in this setting. Moreover, our results reveal a significant negative correlation between 24 h UFC levels and the CP score, as well as CRP levels. No association was observed between 24 h UFC and 5-day treatment failure or 6-week mortality. However, patients who experienced rebleeding, infection, or death (or any combination of these events) exhibited lower levels of 24 h UFC versus those who remained free of these events.

In the last twenty years, there has been substantial research on RAI in chronic liver disease, and the results have shown that this condition is very common among cirrhotic patients who are critically ill, such as those experiencing sepsis, septic shock, and AVB [9,10,11,12,13,14,15,16,17,18,19,20,21,55,56,57]. Emerging data suggest the existence of RAI in both critical and non-critical states. However, a worsening synthetic liver function is a predictive marker for RAI development, and these patients should be assessed for impaired adrenal function periodically, mainly as a marker of disease severity. While the underlying mechanisms of adrenal dysfunction in critically ill cirrhotic patients share similarities with conditions like septic shock [58,59], liver dysfunction itself appears to contribute to the development of adrenal insufficiency in non-critically ill individuals with cirrhosis [60]. Accumulating evidence indicates that the baseline adrenocortical function in liver cirrhosis is suboptimal and characterized by reduced cortisol synthesis and elimination rates. This abnormality becomes more prevalent as liver function deteriorates. The timely identification of adrenal insufficiency is crucial for cirrhotic patients [14], as it is considered an early predictor of mortality in this population [47].

The use of 24 h UFC for the estimation of adrenal function could possibly overcome the limitations of total serum and salivary cortisol measurements, as this approach is not affected by either oral cavity hygiene or liver metabolism. In addition, 24 h urine measurement seems more efficient as it integrates with daily cortisol production and is not influenced by CBG or albumin. In the present study, we showed a novel significant association between 24 h UFC levels and free plasma cortisol levels, highlighting its use as a possible indicator of adrenal cortisol production in patients with liver cirrhosis and variceal bleeding. Our recent study demonstrates that the use of 24 h UFC is an easy, trustworthy, and noninvasive alternative method of determining adrenal cortisol production instead of serum-free cortisol or salivary cortisol in stable patients with liver cirrhosis [46]. Moreover, 24h UFC has been characterized as a dependable indicator for diagnosing hypercortisolism [61]. Another important finding of the present study was the role of low 24 h UFC levels as an indicator of liver disease progression. Specifically, significant and negative associations were observed between 24 h UFC with CP score and CRP. Research on the prognostic significance of different cortisol forms on liver disease progression has shown conflicting results [46,62,63]. Thavenot et al. reported that high free cortisol levels are strongly associated with CRP levels, implying that systemic inflammation can cause a significant increase in cortisol levels [62]. On the other hand, our previous study demonstrated significant differences in the development of decompensation between stable cirrhotic patients who presented with low versus high levels of 24 h UFC [46]. Aligning with our study findings, a recent investigation revealed a decline in adrenal total cortisol levels corresponding to advancing clinical substages of advanced chronic liver disease (ACLD) [63]. This indicates the suppression of the HPA axis, particularly in individuals with ACLD. Additionally, this study demonstrated that increased systemic inflammation and bile acid levels might contribute to the suppression of pituitary–adrenal cortisol signaling [63]. Notably, lower levels of total cortisol and free cortisol are linked to unfavorable clinical outcomes, signifying an elevated risk for subsequent bacterial infections and further decompensation [63]. 

Adrenal insufficiency may contribute to hemodynamic instability and a diminished response to vasoactive medications, much like other critical conditions [11]. In the case of variceal bleeding, adrenal insufficiency could potentially increase the likelihood of rebleeding or failure to control bleeding, leading to reduced survival rates. Triantos et al. showed that increased calculated free cortisol levels were a strong predictor of short-term mortality in patients with AVB [20]. In the present study, no correlation was documented between 24 h UFC levels and the outcomes of 6-week mortality and 5-day treatment failure. The only independent risk factor for the 5-day treatment failure in our study was the presence of PVT. PVT serves as an independent risk factor for 5-day treatment failure as it can elevate the resistance to portal inflow, further compromising the hepatic blood flow [64,65]. There is no report in the literature regarding the use of urinary cortisol as a surrogate marker for the evaluation of these outcomes in the setting of variceal bleeding. Michailidou et al. indicated that 24 h UFC levels represent a prognostic marker for mortality as higher levels of 24 h UFC are independently correlated to stable cirrhosis survival [46]. A novel finding of our study, which is consistent with the abovementioned results, is that patients who experienced rebleeding, infection, or death (or any combination of these events) exhibited lower levels of 24 h UFC versus those who remained free of these events, indicating that the absence of a significant association between urine cortisol and bleeding outcomes in the univariate analysis might be due to a type 2 error. The prognostic role of other cortisol forms in the context of adrenal insufficiency has been investigated in patients with liver disease [13,46,62,63,66,67]; however, the existing data are conflicting. In line with our study, Fede et al., via evaluating the free cortisol or total cortisol levels, revealed no correlation between adrenal dysfunction and mortality or liver transplantation outcomes [66]. Thevenot et al. documented that increased stimulated free cortisol levels were linked to reduced survival rates among stable cirrhotic patients who were hospitalized for routine cirrhosis check-ups [62]. On the other hand, a study that included hemodynamically stable cirrhotic outpatients demonstrated a significant association between mortality and adrenal dysfunction defined as stimulated free cortisol < 33 nmol/L [13]. Reduced total cortisol production has been linked to an increased occurrence of type 1 hepatorenal syndrome, severe sepsis, and elevated short-term mortality in non-critically ill cirrhotic patients admitted for acute decompensation [67]. Lastly, decreased levels of total cortisol have been related to acute-on-chronic liver failure and liver-related mortality in stable patients with ACLD [63]. These conflicting results could potentially be addressed by considering the inclusion of severely critically ill patients in some of these studies or if the prognostic significance of cortisol is explored in the context of adrenal insufficiency [13,62,66,67]. Additional research involving larger sample sizes and repeated cortisol measurements is needed to elucidate the prognostic significance of 24 h UFC, particularly in distinguishing between various categories of cirrhotic patients, including those who are compensated or decompensated without variceal bleeding and those with variceal bleeding. Considering that 24 h UFC presents a large intra-individual variability in healthy conditions, the need for further investigation becomes stronger in the presence of acute diseases.

A rationale for the existence of hepatoadrenal syndrome in patients with end-stage cirrhosis still exists. Evidence indicates that inflammation can trigger abnormal endothelial function, which, in turn, impairs cortisol synthesis [68]. Consequently, in individuals with compensated cirrhosis, although adrenal insufficiency may be present as a latent condition, a proinflammatory microenvironment appropriately activates the HPA axis, resulting in higher cortisol levels [69]. However, when stress becomes more severe (accompanied by the excessive production of pro-inflammatory cytokines), steroid resistance may develop, leading to the insufficient production of cortisol [69]. Triantos et al. confirmed the high prevalence of RAI in patients with cirrhosis during acute gastrointestinal bleeding [21]. Patients with RAI bear an increased risk of treatment failure and increased mortality rates compared to patients with normal adrenal function. In parallel, patients with RAI present an elevated number of infections both at admission and hospitalization. Studies have demonstrated that among noncritically and critically ill cirrhotics, patients with RAI have an increased probability of renal failure, sepsis, and mortality. Moreover, increased baseline cortisol levels indicate which patients are susceptible to developing RAI, implying the existence of adrenal exhaustion in this content [19]. Thus, the worse prognosis of a patient with RAI might be associated with various parameters [70]. RAI is linked to a significant decline in circulatory function, which could potentially lead to treatment failure in patients with AVB. Moreover, RAI in the setting of AVB might increase the risk of early clot dislodgement by hindering the compensatory vasoconstriction in the splanchnic region, which typically occurs in response to hypovolemia. Collectively, these findings suggest that individuals with cirrhosis and variceal hemorrhage exhibit various disruptions in cortisol regulation. Several mechanisms underlie these disruptions, including the release of inflammatory cytokines that interfere with the HPA axis, adrenal hypoperfusion due to circulatory dysfunction, and reduced cholesterol production by the liver.

Our study comes with certain limitations. More importantly, not all multivariate analyses could be performed due to the relatively small sample size and the subsequent low number of events. However, all patients were prospectively included in the analysis, and we did not have any missing data to handle. Another limitation is the mono-centric design, and this highlights the need for the confirmation of the present findings by other scientific centers.

## 5. Conclusions

In conclusion, the present study suggests the use of 24 h UFC as a reliable and noninvasive surrogate marker of adrenal cortisol production in patients with liver cirrhosis and variceal bleeding as it is adequately associated with plasma-free cortisol levels, overcoming the disadvantages of other cortisol forms. Moreover, our findings indicate that low levels of 24 h UFC impose a risk factor for patients with liver cirrhosis and variceal bleeding. Lastly, although 24 h UFC was not found to be an independent prognostic marker for 5-day treatment failure and 6-week mortality, 24 h UFC levels were significantly lower in patients who suffered from rebleeding, infection, death, or a combination of these events compared to patients who did not, demonstrating the potential clinical significance of 24 h UFC for this cohort. The conduction of further studies is deemed necessary to provide robust evidence for the role of 24 h UFC in the evaluation of HPA axis activity.

## Figures and Tables

**Table 1:** Baseline characteristics.

	Median (IQR)
Age (years)	56.5 (47.5–68.5)
Hemoglobin (g/dL)	9.5 (7.7–10.4)
WBC count (cells/μL)	7900 (5500–11,800)
Platelet count (cells/μL)	85,000 (65,000–118,000)
INR	1.4 (1.3–1.6)
Total bilirubin (mg/dL)	2.0 (1.3–2.9)
Albumin (g/dL)	3.2 (3.0–3.4)
Creatinine (mg/dL)	0.8 (0.7–1.0)
Serum sodium (mmol/L)	139.0 (135.6–140.8)
Child–Pugh score	7 (6–9)
MELD score	12 (10–13.8)
	N (%)
Sex (M/F)	32/8 (80/20)
Etiology of liver cirrhosis (viral/alcoholic/NAFLD/other)	10/17/1/12 (25.0/42.5/2.5/30.0)
Previous variceal bleeding	13 (32.5)
Beta blockers use	19 (47.5)
HCC at baseline	7 (17.5)
Portal vein thrombosis	4 (10)
Child–Pugh stage (A/B/C)	11/21/5 (27.5/52.5/12.5)

Abbreviations: IQR, interquartile range; WBC, white blood cells; INR, international normalized ratio; MELD, model for end-stage liver disease; N, number of patients; M, male; F, female; NAFLD, non-alcoholic fatty liver disease; HCC, hepatocellular carcinoma.

**Table 2:** Adrenal function parameters.

	Median (IQR)
Total serum cortisol (μg/dL)	18.7 (12.0–25.26)
Salivary cortisol (μg/dL)	0.59 (0.38–1.46)
Cortisol-binding globulin (μg/mL)	27.66 (23.12–30.91)
Free plasma cortisol (μg/dL)	5.72 (3.68–13.84)
24 h urinary free cortisol (mg/24 h)	115.76 (38.57–447.07)

Abbreviations: IQR, interquartile range.

## Data Availability

The data presented in this study are available on request from the corresponding author. The data are not publicly available as they involve human subjects, and their confidentiality and ethical considerations must be respected.

## Supplementary Materials

The following supporting information can be downloaded at: https://www.mdpi.com/article/10.3390/medicina59122112/s1, Table S1: spearman’s correlations (r) between total serum cortisol, free plasma cortisol, salivary cortisol, cortisol binding globulin and 24h urinary free cortisol; Table S2: spearman’s correlations (r) between adrenal function parameters and clinical parameters; Table S3: univariate and multivariate analyses of factors predicting 6-week mortality in patients with variceal bleeding; Table S4: univariate and multivariate analyses of factors predicting 5-day treatment failure in patients with variceal bleeding; Table S5: comparison of cortisol levels between patients who experienced rebleeding or infection or death (or any combination of these events) vs those who remained free of these events.
