# Supplementary material for: Low Urinary Free Cortisol as a Risk Factor for Patients with Variceal Bleeding
Source: Medicina (Kaunas). 2023 Dec 2;59(12):2112. doi: 10.3390/medicina59122112 (PMC10744924; doi:10.3390/medicina59122112)
Supplement: Supplementary file 1 [file medicina-59-02112-s001.zip › medicina-2711450-SI.pdf]

Supplementary Table S1. Spearman's correlations (r) between total serum cortisol, free plasma cortisol, salivary cortisol, cortisol binding globulin and 24h urinary free cortisol.

	**TSC (μg/dl)**	**SC (μg/dl)**	**CBG (μg/ml)**	**FC (μg/dl)**	**24h UFC (mg/24h)**
**TSC (μg/dl)**		**0.356 (P = 0.049)**	0.301 (P = NS)	**0.672 (P <0.001)**	0.237 (P=NS)
**SC (μg/dl)**	**0.356 (P = 0.049)**		-0.079 (P = NS)	0.049 (P = NS)	0.027 (P = NS)
**CBG (μg/ml)**	0.301 (P = NS)	-0.079 (P = NS)		**0.449 (P = 0.009)**	- 0.014 (P = NS)
**FC (μg/dl)**	**0.672 (P <0.001)**	0.049 (P = NS)	**0.449 (P = 0.009)**		**0.362 (P = 0.042)**
**24h UFC (mg/24h)**	0.237 (P=NS)	0.027 (P = NS)	- 0.141 (P = NS)	**0.362 (P = 0.042)**	

Abbreviations: TSC, total serum cortisol; FC, free plasma cortisol; SC, salivary cortisol; CBG, cortisol binding globulin; 24h UFC, 24h urinary free cortisol; NS, not significant.

Supplementary Table S2. Spearman's correlations (r) between adrenal function parameters and clinical parameters

		**MELD score**	**CP score**	**HCT**	**PLTs**	**Cr**	**Alb**	**Tbil**	**CRP**
**CBG**	r	0.021	0.308	0.198	-0.039	0.104	0.190	0.182	-0.050
	p-value	0.909	0.086	0.262	0.827	0.557	0.290	0.302	0.824
**FC**	r	-0.083	0.036	0.024	-0.103	0.275	0.066	-0.047	-0.193
	p-value	0.657	0.844	0.893	0.567	0.122	0.719	0.794	0.389
**TSC**	r	-0.345	-0.137	-.0116	0.184	0.294	-0.014	-0.358	-0.118
	p-value	0.062	0.461	0.514	0.297	0.091	0.938	**0.038**	0.600
**SC**	r	0.109	0.216	-0.363	0.162	0.058	-0.472	0.062	-0.027
	p-value	0.575	0.251	**0.041**	0.374	0.751	**0.007**	0.737	0.901
**24h UFC**	r	0.115	-0.360	0.093	-0.009	0.055	0.078	-0.165	-0.439
	p-value	0.543	**0.047**	0.601	0.961	0.758	0.667	0.350	**0.041**

Abbreviations: HCT, hematocrit; PLTs, platelets; Cr, creatinine; Alb, albumin, Tbil, total bilirubin; CRP, C reactive protein; CBG, cortisol binding globulin; FC, free plasma cortisol; TSC, total serum cortisol; SC, salivary cortisol; 24h UFC, 24h urinary free cortisol

Supplementary Table S3. Univariate and multivariate analyses of factors predicting 6-week mortality in patients with variceal bleeding

	HR (95% CI)	P value	HR (95% CI)	P value
**Age**	1.001 (0.93-1.07)	NS		
**Previous variceal bleeding**	0.46 (0.05-4.58)	NS		
**B-blockers use**	0.75 (0.11-5.07)	NS		
**HCC**	0.26 (0.03-1.96)	NS		
**Portal vein thrombosis**	10.67 (1.08-105.28)	**0.043**		
**INR**	1.77 (0.37-8.41)	NS		
**Creatinine**	3.77 (0.65-22.02)	NS		
**Albumin**	6.39 (0.55-74.90)	NS		
**Total Bilirubin**	1.43 (0.59-3.39)	NS		
**Sodium**	0.88 (0.69-1.12)	NS		
**Ascites**	2.04 (0.29-13.85)	NS		
**Encephalopathy**	0.39 (0.03-4.68)	NS		
**CP stage C**	30 (1.41-638.15)	**0.029**	27.00 (1.26-578.35)	**0.035**
**CP score**	2.20 (0.97-4.99)	0.06		
**MELD score**	1.23 (0.995-1.53)	0.056		
**In-hospital infection**	10.67 (1.08-105.28)	**0.043**		
**Active bleeding during endoscopy**	0.46 (0.07-3.27)	NS		
**Rebleeding**	24.00 (2.43-236.89)	**0.007**		
**FC (μg/dl)**	0.99 (0.88-1.13)	NS		
**CBG (μg/ml)**	0.86 (0.72-1.01)	0.065		
**TSC (μg/dl)**	1.01 (0.94-1.10)	NS		
**SC (μg/dl)**	1.16 (0.69-1.95)	NS		
**24h UFC (mg/24h)**	1.00 (0.99-1.00)	NS		

Abbreviations: HR, hazard ratio; CI, confidence interval; NS, not significant; HCC, hepatocellular carcinoma; INR, International normalized ratio; CP, Child-Pugh; MELD, Model for End-stage Liver Disease; FC, free plasma cortisol; CBG, cortisol binding globulin; TSC, total serum cortisol; SC, salivary cortisol; 24h UFC, 24h urinary free cortisol

Supplementary Table S4. Univariate and multivariate analyses of factors predicting 5-day treatment failure in patients with variceal bleeding

	**Univariate Analysis**		**Multivariate Analysis**	
**Variable**	**OR (95% CI)**	**P value**	**OR (95% CI)**	**P value**
**Age**	0.98 (0.92-1.05)	NS		
**Sex (male)**	1.00 (0.10-10.41)	NS		
**B-blockers use**	1.42 (0.21-9.55)	NS		
**HCC**	0.83 (0.08-8.77)	NS		
**Portal vein thrombosis**	51.00 (3.51-740.134)	**0.004**	39.00 (2.67-569.67)	**0.007**
**INR**	0.97 (0.15-6.22)	NS		
**Creatinine**	0.10 (0.02-6.07)	NS		
**Albumin**	0.16 (0.02-1.34)	NS		
**Total Bilirubin**	1.11(0.48-2.56)	NS		
**Sodium**	1.13 (0.90-1.42)	NS		
**CP stage C**	13.33 (1.01-196.37)	**0.05**		
**CP score**	2.20 (0.97-4.99)	0.06		
**MELD score**	0.89 (0.49-1.61)	NS		
**In-hospital infection**	0.14 (0.02-1.20)	NS		
**Active bleeding during endoscopy**	1.39 (0.14-14.07)	NS		
**FC (μg/dl)**	1.01 (0.88-1.14)	NS		
**CBG (μg/ml)**	0.88 (0.75-1.03)	0.065		
**TSC (μg/dl)**	1.02 (0.94-1.10)	NS		
**SC (μg/dl)**	1.19 (0.70-2.03)	NS		

**24h UFC (mg/24h)**	1.00 (0.99-1.01)	NS		

Abbreviations: HR, hazard ratio; CI, confidence interval; NS, not significant; HCC, hepatocellular carcinoma; INR, International normalized ratio; MELD, Model for End-stage Liver Disease; FC, free plasma cortisol; CBG, cortisol binding globulin; TSC, total serum cortisol; SC, salivary cortisol; 24h UFC, 24h urinary free cortisol

Supplementary Table S5. Comparison of cortisol levels between patients who experienced rebleeding or infection or death (or any combination of these events) vs those who remained free of these events.

	**Composite endpoint**	**Median (IQR)**	**p-value**
**FC**	Yes	4.40 (4.03-10.31)	P=0.9
	No	6.54 (3.65-13.76)	
**CBG**	Yes	28.97 (28.12-35.29)	P=0.12
	No	26.34 (23.29-29.78)	
**TSC**	Yes	13.79 (12.06-21.43)	P=0.641
	No	20.91 (12.03-25.49)	
**SC**	Yes	0.60 (0.33-0.82)	P=0.726
	No	0.50 (0.39-1.58)	
**24h UFC**	Yes	39.40 (19.13-57.40)	**P=0.022**
	No	163.38 (43.03-449.63)	

Abbreviations: IQR, interquartile range; FC, free plasma cortisol; CBG, cortisol binding globulin; TSC, total serum cortisol; SC, salivary cortisol; 24h UFC, 24h urinary free cortisol
